# Molecular Approaches for Food Protein Allergenicity Assessment and the Diagnosis and Treatment of Food Allergies

**DOI:** 10.3390/foods12061205

**Published:** 2023-03-12

**Authors:** Daniel Lozano-Ojalvo, Sara Benedé

**Affiliations:** 1 Precision Immunology Institute, Icahn School of Medicine at Mount Sinai, New York, NY 10029, USA; 2 Instituto de Investigación en Ciencias de la Alimentación (CIAL, CSIC), 28049 Madrid, Spain; 3 Departamento de Inmunología, Oftalmología y Otorrinolaringología, Facultad de Medicina, Universidad Complutense de Madrid, 28040 Madrid, Spain

Food allergy, an adverse immune reaction triggered by commonly innocuous food proteins, is a health problem that affects millions of people worldwide (around 10% of the global population), and the most recent reports suggest its increasing progression [1,2]. Most of the allergic responses have been associated with milk, egg, peanut, tree nuts, soy, wheat, fish, and shellfish. Food allergy has been associated with both IgE- and non-IgE-mediated immune responses. In IgE-mediated food allergies, antigen-presenting cells capture, process, and present digested food proteins to T helper (Th) cells priming their differentiation towards Th2 cells and inducing the generation of allergen-specific IgE antibodies by B cell-derived plasma cells that bind to the high-affinity IgE receptor (FcεRI) placed on mast cells and basophils. Subsequent allergen exposure generates the crosslinking of IgE antibodies bound on the surface of mast cells and basophils that induces their degranulation and the development of anaphylactic symptoms in sensitized subjects [3]. Although the physiopathology of non-IgE-mediated food allergies is not fully understood, the relevance of Th2-mediated responses triggered by food allergens has been reported as cause of many gastrointestinal diseases including eosinophilic esophagitis (EoE), food protein-induced allergic proctocolitis (FPIAP), food protein-induced enteropathy (FPE), and food protein-induced enterocolitis syndrome (FPIES). This Special Issue is dedicated to the recent progress in molecular approaches that advances knowledge in the assessment of the allergenic potential of food components, as well as in the diagnosis, prognosis, and treatment of food allergies.

Despite the increasing knowledge generated for the structural characteristics of food allergens, the involvement of the food matrix and processing in protein allergenicity need to be extended. The inclusion of new ingredients in the food industry such as emulsifiers, stabilizers, and thickening agents can also be a source of allergenic compounds in the final products offered to consumers. Pectin, a dietary fiber widely used in the food industry as a gelling agent, is mainly obtained from fruits such as peach, apples, or lemons. Anaphylactic reactions have been reported after the consumption of pectin-supplemented foods, frequently associated with contaminant non-specific lipid-transfer proteins (nsLTPs) which could impair the use of pectins in nsLTP allergic patients. Steigerwald et al. have evaluated the presence of the nsLTP Pru p 3 in commercial pectins showing that the potential residual content of this allergen is below the threshold to induce anaphylactic reactions in nsLTP allergic patients [4]. The interaction of food proteins with other components of the matrix can alter their allergenicity. In this context, the ability of various natural products found in apples such as flavonoids, glutathione, and glutathione disulfide to bind the major allergen of this fruit Mal d 1 have been studied using microscale thermophoresis by Chebib and Schwab [5]. Authors found that while flavonoids are bound to Mal d 1 via hydrophobic and polar interactions, glutathione binding to this allergen occurs by hydrophilic hydrogen bonds and van der Waal forces, suggesting differential modification of the allergenicity of Mal d 1 [5]. Processing food products can induce modifications in the proteins not only by modifying their allergenicity but also by changing their digestibility. For instance, Benedé et al. have studied how heat treatment of food allergens can induce structural modifications that alter their digestibility, affecting the biological interaction with epithelial and immune cells. The differential response of Caco-2 cells induced by the peptides generated after digestion of raw and heat-treated egg white allergens has been studied [6]. Results showed that while digestion of raw Gal d II promoted the expression of pro-allergenic (TSLP) and pro-inflammatory (IL6) cytokines in Caco-2 cells, digestion of the heat-treated allergen reduces the expression of these cytokines, suggesting that the heating process might reduce the release of Gal d II-derived allergenic epitopes [6]. The study of the clinical relevance of the individual contribution of proteins contained in the food to its whole allergenicity is a critical factor in the evaluation of the risk assessment and the development of safer methods for allergenic evaluation. In this sense, Valbuena et al. have evaluated the demographic variables, clinical characteristics, individual contribution of hazelnut allergens (Cor a 1, Cor a 8, Cor a 9, Cor a 11, and Cor a 14), and biomarkers that can be associated with severe reactions during the hazelnut oral challenge test as well as skin prick tests, specific IgE levels, and component-resolved diagnosis (CRD) [7]. This study has shown that the severity of the anaphylactic reactions is associated with higher levels of Cor a 11- and Cor a 14-specific IgE, and that the use of the CRD is a useful tool to identify patients at high risk of developing severe symptoms [7].

The multiple clinical presentations of food allergies and the different structural characteristics of food allergens have posed a relevant challenge in the diagnosis of this disease. The development of new molecular methods that could improve diagnostic approaches to be more accurate and able to discriminate between asymptomatic and clinically significant sensitization to foods is still needed. For instance, a deeper characterization of allergen structural features can help to identify similarities between allergens and cross-reactivity processes. Bueno-Díaz et al. have assessed how the structural features of 2S albumins affect their immunogenic capacity [8]. They evaluated the molecular and structural characteristics of several 2S albumins isolated from plant-derived extracts by chromatographic methods and they determined the allergenicity of these proteins via immunoblotting. Results established a marked correlation between 2S structural features and their allergenic potential, including cross-reactivity [8]. In this context, the molecular characterization of food allergens is essential for the improvement of diagnosis and prognosis approaches, as shown in this Special Issue for α-Gal-related meat allergy [9] and the oyster allergen Cra g 1 [10]. α-Gal present in tick saliva induces the sensitization to this allergen and generates the production of specific IgG1 and/or IgE, meaning it is possible that the more times a person is bitten by ticks, the higher the probability is of inducing class switching from α-Gal-specific IgG to IgE. Authors quantified circulating levels of specific IgG and demonstrated that when people bitten by ticks had levels higher than 40 µg/mL of α-Gal-specific IgG1, their risk of developing IgE-mediated allergy was 35%, indicating that serum levels of specific IgG against α-Gal might be a prognostic marker of meat allergy [9]. Diagnosis of oyster allergy, a product usually consumed raw, is limited due to the insufficient characterization of the allergens contained in this food and the poor cross-reactivity existing with other shellfish that are regularly heat-cooked. Nugraha et al. have evaluated the IgE sensitization profiles of oyster allergic subjects in order to assess the allergenic potential of the major proteins contained in this food [10]. Tropomyosin Cra g 1 was identified as the major allergen recognized by circulating specific IgE and revealed a certain level of cross-reactivity of tropomyosin from prawn and dust mite, potentially based on the existence of preserved IgE-binding epitopes. This study suggests that recombinant Cra g 1 could be used for component-resolved diagnostics and immunotherapeutic strategies [10].

To date, oral food challenge (OFC) with the triggering food remains the gold standard for the diagnosis of food allergies, but the many risks and drawbacks associated with this approach have resulted in common scientific efforts for improving of IgE-based assays (specific IgE levels, skin prick test, and basophil activation test) and implementing safer and less invasive diagnostic tests. The oral mucosa plays an important role in the generation of tolerogenic immune responses to foods. However, oral mucosa remodeling is associated with epithelial barrier disruption and that induces inflammatory signals. Gómez-Casado et al. have reviewed how the structure and immunological features of the oral mucosa allows us to assess the onset, progression, and outcome of a variety of inflammatory diseases and allergies based on local mucosa-associated immune responses that are connected to the systemic immune system [11]. Recent studies have evaluated the ability of mucosal biomarkers to identify food allergic patients. The easy accessibility to the oral cavity could facilitate diagnosis and help to understand disease progression in complex allergic syndromes, such as nsLPT-mediated food allergy. Despite the significant lack of description of the mechanisms underlying the local immune response in the oral mucosa, current knowledge suggests that it might be helpful not only to depict immune diseases, but also to understand treatment efficacy and the development of novel therapeutic strategies [11].

Treatment of food allergies has become the most challenging matter in the field. In subjects with IgE-mediated food allergies, the avoidance of the triggering food has been the only approach for many years. However, the inadvertent and involuntary intake of the allergens has been a cause of a large number of serious anaphylactic reactions. For this reason, many therapeutic strategies are under investigation including allergen-specific immunotherapies (IT), different routes of administration, new approaches based on hypoallergens and nanoparticles as well as biologics and microbiota-targeted strategies [1]. Allergen-specific IT in which controlled quantities of an allergen are given to a patient in order to induce tolerance has been proposed as a reliable alternative approach for food allergy treatment. The first drug approved by the Food and Drug Administration for the treatment of food allergy was Palforzia, an oral IT for the treatment of peanut allergy in patients aged from 4 to 17 years old with a confirmed diagnosis. However, this drug mainly increases the amount of consumed peanut protein instead of resolving the actual disease. Therefore, there is a need to develop safe and long-term effective therapies for food allergy. In this regard, Mayorga et al. have reviewed the immune mechanisms underlying the current explored strategies for the treatment of IgE-mediated food allergies and have discussed promising therapeutic approaches based on hypoallergenic agents for IT, monoclonal antibodies, and probiotics/prebiotics/synbiotics [1]. Non-IgE-mediated food allergies are more uncommon than IgE-mediated ones, and their mechanisms, diagnosis, and treatment remain largely unknown. Zubeldia-Varela et al. have examined the existing therapeutical options and research strategies for some non-IgE-mediated food allergies including FPIAP, FPE, and FPIES in this Special Issue [12]. In this review, the potential molecular and omics methods that have been used for a better understanding of the pathophysiological mechanisms involved in non-IgE-mediated food allergies have been discussed, including the identification of diagnostic and/or prognostic biomarkers as well as their use as potential therapeutical targets. Therefore, despite the important efforts that have been made in improving therapies for food allergies, including innovative approaches mainly focusing on efficacy and safety, there is an urgent need to develop a set of basic and clinical results to help in the diagnosis and treatment of this disease.

In short, the original articles and reviews collected in this Special Issue, submitted by internationally recognized research teams, cover various areas in the field of food allergy, attempting to shed more light on the different relevant facets of this topic with a particular focus on the food protein allergenicity assessment and the diagnosis and treatment of food allergies.
